# Exercise Interventions Delivered Through Telehealth to Improve Physical Functioning for Older Adults with Frailty, Cognitive, or Mobility Disability: A Systematic Review and Meta-Analysis

**DOI:** 10.1089/tmj.2023.0177

**Published:** 2024-04-08

**Authors:** Rik Dawson, Juliana S. Oliveira, Wing S. Kwok, Marte Bratland, Ian Matthew Rajendran, Ajith Srinivasan, Chun Yin Chu, Marina B. Pinheiro, Leanne Hassett, Catherine Sherrington

**Affiliations:** ^1^ Sydney Musculoskeletal Health, Insititue for Musculoskeletal Health, The University of Sydney, Sydney Local Health District, Gadigal Country, Sydney, Australia.; ^2^ Sydney School of Health Sciences, Faculty of Medicine and Health, The University of Sydney, Gadigal Country, Sydney, Australia.

**Keywords:** *physical therapy*, *aged care*, *rehabilitation*, *telemedicine*, *telehealth*

## Abstract

**Introductions:**

*This study assessed the effects of telehealth-delivered exercise interventions on physical functioning for older adults and explored implementation measures related to program delivery.*

**Methods:**

*We conducted a systematic review of studies investigating effects of exercise interventions delivered through telehealth in adults 60+ years of age with frailty, mobility, or cognitive disability on mobility, strength, balance, falls, and quality of life (QoL). Electronic databases (MEDLINE, CINAHL, SPORTSDiscus, and Physiotherapy Evidence Database) were searched from inception until May 2022. Evidence certainty was assessed with Grading of Recommendations, Assessment, Development, and Evaluation and meta-analysis summarized study effects.*

**Results:**

*A total of 11 studies were included, 5 randomized controlled trials, 2 pilot studies, and 4 feasibility studies. The overall certainty of evidence was rated as “low” or “very low.” Pooled between-group differences were not statistically significant, but effect sizes suggested that telehealth produced a moderate improvement on mobility (*n* = 5 studies; standardized mean difference [SMD] = 0.63; 95% confidence interval [CI] = −0.25 to 1.51;* p* = 0.000,* I*^2^ = 86%) and strength (*n* = 4; SMD = 0.73; 95% CI = −0.10 to 1.56;* p* = 0.000,* I*^2^ = 84%), a small improvement on balance (*n* = 3; SMD = 0.40; 95% CI = −035 to 1.15;* p* = 0.012,* I*^2^ = 78%), and no effect on QoL. Analysis of implementation measures suggested telehealth to be feasible in this population, given high rates of acceptability and adherence with minimal safety concerns.*

**Discussion:**

*Telehealth may provide small to moderate benefits on a range of physical outcomes and appears to be well received in aged care populations.*

## Introduction

Older adults, 60 years of age and older, are the most sedentary age group in our community.^1^ Aged care service recipients, who usually have frailty, and/or mobility or cognitive disability, are particularly inactive.^2^ Physical inactivity is associated with negative health outcomes such as increased risk of falls, frailty, mobility disability, and death.^3^ Exercise interventions have been found to improve mobility and reduce falls in frail community-dwelling older adults and in aged care populations, who have high levels of mobility and cognitive disability.^4–6^ The Australian 2021 Royal Commission into Safety and Quality in Aged Care identified falls and mobility decline as a serious problem in residential aged care, exacerbated by poor access to exercise health professionals such as physiotherapists.^7^ Similar issues have been identified in other countries.^8^

World Health Organization has defined telehealth as the delivery of health care services, where patients and providers who are separated by distance use information communication technologies to diagnose and treat diseases and injuries.^9^ Telehealth is a rapidly growing service delivery model that could enhance access and facilitate delivery of exercise programs to older adults receiving aged care services in their home or in residential care. Uptake of telehealth has been accelerated in response to the COVID-19 pandemic, especially in aged care settings.^10^

Little is known about the effectiveness of telehealth use to deliver exercise programs in aged care settings or how best to implement telehealth exercise in this complex population. A recent rapid review investigating the use of websites and apps to assist older adults engage with balance and strength training found low to moderate evidence that the use of digital technology improved physical activity and reduced fall risk.^11^ However, this review excluded studies that targeted aged care populations. A discrete choice experiment in 2017 concluded that telehealth was acceptable among older adults receiving rehabilitation, but excluded aged care populations.^12^

This systematic review sought to investigate the use of telehealth exercise for older adults who are receiving aged care services or have frailty, mobility, or cognitive disability. This review aimed to summarize (1) the effects of exercise interventions delivered through telehealth on mobility, strength, balance, falls, and quality of life (QoL) and (2) implementation outcomes and determinants related to the delivery of telehealth exercise programs in this population.

## Methods

Our systematic review with meta-analysis followed the methods described in the Cochrane Handbook for Systematic Reviews of Interventions and the Preferred Reporting Items for Systematic Reviews and Meta-Analyses (PRISMA) guidelines.^13,14^ This review was registered through the PROSPERO international prospective register of systematic reviews on May 1, 2022 (CRD42022322469).

### SEARCH STRATEGY

Optimized searches were completed through four electronic databases (MEDLINE, CINAHL, SPORTDiscus, and Physiotherapy Evidence Database [PEDro]) from inception to May 1, 2022. Four reviewers (I.M.R., C.Y.C., A.S., and M.B.) searched one database each. Keywords, MeSH, and other index terms were used to construct the search strategy (see online *Supplementary Table S1* for examples of search terms). Articles sourced by hand searching were included. Articles were independently screened in two stages: screening of title and abstracts and screening of full-text articles by two pairs of reviewers (I.M.R./A.S. or C.Y.C./M.B.) using the eligibility criteria. Disagreements regarding the eligibility of studies were resolved through discussion. Conference abstracts and dissertations that reported data suitable for analysis were included.

### ELIGIBILITY CRITERIA

#### Type of study

For our first aim, we included randomized controlled trials (RCTs), and for our second aim, we also included quasi-randomized, feasibility, and qualitative studies.

#### Participants

We included studies investigating participants with a mean age of 60+ years with frailty, mobility, or cognitive disability, or who were aged care service users. Any type of health condition was included. Studies involving participants being treated in hospital were excluded as these participants receive more support than older people receiving aged care services in their home or in residential aged care.

#### Interventions

Studies were included if they evaluated exercise interventions delivered through synchronous telehealth (virtual interactions between a participant and a health professional that occurs in real time) or asynchronous telehealth (sharing of data, educational materials, or online programs to assist a participant to exercise at a time of their choosing) that aimed to increase balance, strength, and/or physical functioning.^15^ We excluded studies involving no telehealth, that did not include participants with frailty, mobility, or cognitive disability or aged care service users, or involved only wearable technology. Interventions of any length and any follow-up period were included.

#### Comparator

For our meta-analysis, we included RCTs that compared telehealth to any comparator.

#### Outcomes

Our outcomes to address aim 1 included measures of mobility, balance, strength, falls, and QoL. Outcome data were extracted for baseline and post-intervention periods. Studies were included in meta-analysis if their data were presented as or could be converted into mean/standard deviation (SD) pre-intervention and post-intervention scores to facilitate quantitative pooling. To enable inclusion of as many studies as possible in the meta-analysis, we pooled results across multiple assessment tools for the same outcome.

For studies that reported results for more than one assessment tool for the same outcome, we selected one tool per outcome using a pre-defined order of priority.^16^ The order of priority was as follows: for mobility (Short Physical Performance Battery, Physical Performance Test, Timed Up and Go test, and walking speed); for balance (Berg Balance Scale, 4-Stage balance test, and timed step test); strength (only timed sit to stand was used); and QoL (no priority was required as there was only one tool used in each study). Falls were assessed separately using the risk of falling that is, number of adults who experienced one or more falls and rate of falls that is, falls per person-year.

Outcomes to address aim 2 included any implementation-related outcome and determinant identified through an analysis of studies' results relating to the intervention's reach (proportion of participants who were successfully screened and consented to participate),^17^ feasibility (proportion of participants who completed the follow-up assessment),^18^ adherence (proportion of participants who completed the agreed number of planned intervention sessions),^19^ acceptability (measure of satisfaction), dose (hours of exercise completed over study period), and safety (reporting of adverse events [AE] such as falls and pain directly related to the intervention), as well as barriers and facilitators determined using mixed methods.^20^

### DATA EXTRACTION

A data extraction sheet was developed, pilot-tested, and modified accordingly. For each study, two pairs of investigators (I.M.R./A.S. or C.Y.C./M.B.) extracted the data, and two investigators (R.D./W.S.K.) checked the data. Information extracted from each study comprised a description of participants, details of the intervention, and outcome measures (baseline and at first follow-up). Preintervention and post-intervention scores were used when available. Authors of the included studies were contacted by email if the study reports were incomplete, or data were missing. If the author did not reply, then the available data were used. For our meta-analysis, the pooled difference was calculated as a mean and 95% confidence intervals (CIs) for each outcome in the simple stratified analysis.

### METHODOLOGICAL QUALITY ASSESSMENT FOR RCTS

Data pertaining to the risk of bias were extracted by two pairs of investigators (I.M.R./A.S. or C.Y.C./M.L.) and assessed using the PEDro scale.^21^ The PEDro scale evaluates 11 items: inclusion criteria and source, random allocation, concealed allocation, similarity at baseline, subject blinding, therapist blinding, assessor blinding, completeness of follow-up, intention-to-treat analysis, between-group statistical comparisons, and point measures and variability.^21^ Item 1 refers to external validity and does not contribute to the final score; thus, the final scores ranged from 0 to 10.

### ASSESSMENT OF CERTAINTY OF THE EVIDENCE

The Grading of Recommendations, Assessment, Development, and Evaluation (GRADE) was used to assess the certainty of evidence for the primary outcomes of our meta-analyses for aim 1.^22^ We used the GRADE system for all outcomes, which pooled results from three or more studies. We evaluated the quality of the body of evidence as “High,” “Moderate,” “Low,” or “Very Low” based on the presence or extent of four factors: study limitations, inconsistency of the effect, imprecision, and publication bias.^23^

### DATA ANALYSIS

Meta-analyses were completed using Stata Meta-Analysis software using the random-effects model for each outcome (mobility, strength, balance, and QoL).^24^ We gathered preintervention and post-intervention mean/SD and the sample size per group. We used the controlled trials that compared the telehealth intervention group with either usual care that included no active exercise (four trials)^25–28^ or face-to-face in-person exercises (one trial).^29^ We calculated treatment effects using standardized mean differences (SMDs) (Hedges' g), standardized by post-score SD (or its estimate) with 95% CIs. SMD was calculated using the pre-mean and post-mean and SD or, when this was unavailable, we used the mean change score. Effect sizes were categorized as small (0.2–0.49), medium (0.5–0.79), or large (0.8 or greater).^30^ We visually inspected forest plots for evidence of heterogeneity with consideration of the *I*^2^ and *χ*^2^ tests. For the implementation data, we collected median and range scores and conducted a thematic analysis of the authors' reported barriers and facilitators to implementing telehealth.

## Results

**Fig. 1.**
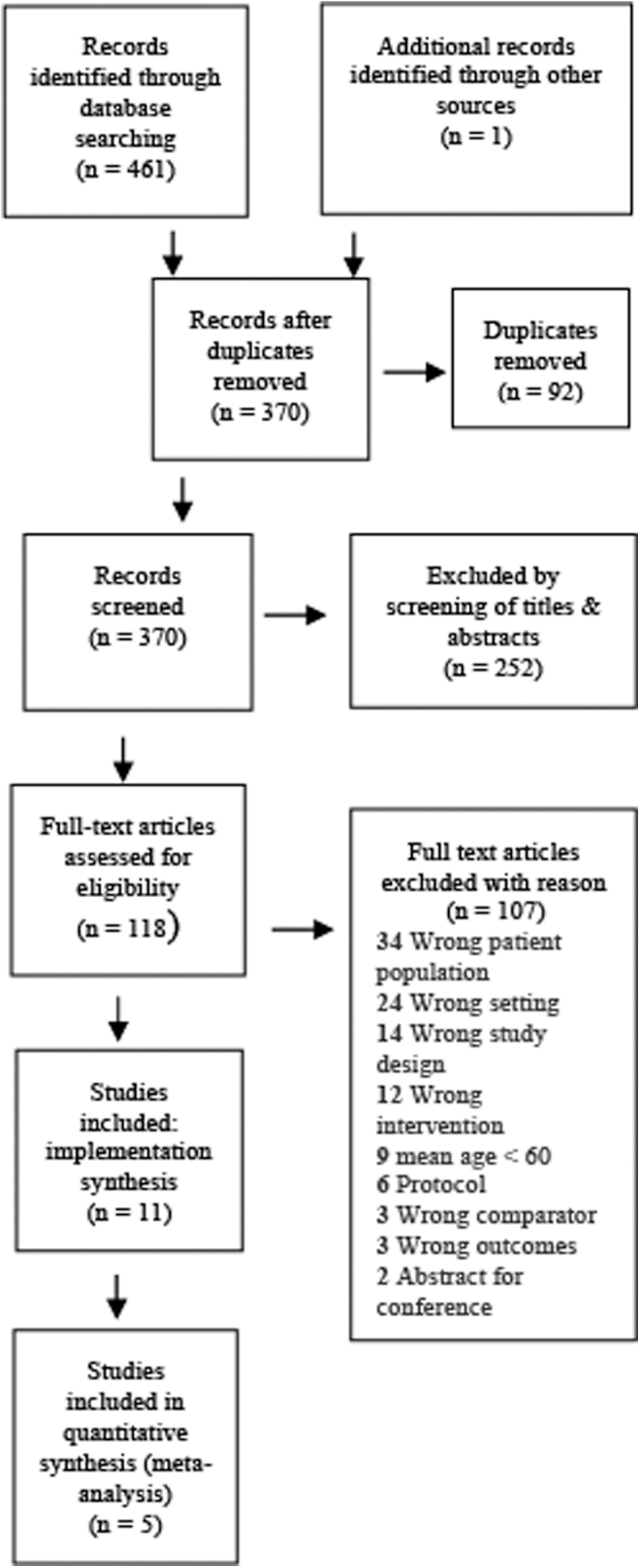
Preferred Reporting Items for Systematic Reviews and Meta-Analyses flow chart.

After duplicates were removed, the electronic search retrieved 370 references. We completed full-text screening on 118 articles. We included 11 studies for the review, which included 5 RCTs, 4 feasibility studies, and 2 pilot studies. The five trials contributed to our meta-analysis (aim 1) and all the studies contributed to our implementation analysis (aim 2). Search results are presented in *Figure 1*.

### TRIAL DESIGN AND PARTICIPANT CHARACTERISTICS

Participant characteristics are displayed in *Table 1*. A total of 546 subjects participated across all studies (302 in controlled studies and 244 in feasibility or pilot studies). Mean age was 77.4 ± 4.7 years and 65% of participants were female. Three studies recruited from residential aged care facilities^27,31,32^ and one study recruited participants receiving aged care services in their home.^28^ Eight studies recruited participants with a mobility disability,^25–28,32–35^ five studies included participants with cognitive impairment,^25–27,31,35^ and three studies recruited frail participants.^25,27,36^ In the controlled studies, two compared telehealth to usual care,^27,28^ with one study compared to usual care plus fall education,^25^ one study compared to seated stretching,^26^ and one study compared to in-person balance training.^29^ The mean PEDro total score of the RCTs was good at 6.4.

**Table 1.** Trial Design and Participant Characteristics (*N* = 11)

AUTHOR (YEAR), COUNTRY, PEDRO SCORE^a^	STUDY DESIGN, LENGTH IN WEEKS	SAMPLE SIZE (***I*** & ***C***)	AGE (YEARS, MEAN ± SD)	FEMALE (%)	SETTING	CONDITION AND SEVERITY	CONTROL	OUTCOMES (END OF INTERVENTION)
Bernard et al. (2009),^31^ Canada, NA	Pilot study, 10 weeks	*n* = 22	81 ± 7	53%	Aged care facility	Comorbidities, mild to moderate CI	No control group	Strength
Bruns et al. (2019),^33^ The Netherlands, NA	Pilot study, 3.7 weeks (median)	*n* = 14	79 IQR = 74–76	64%	Home	Frailty, cancer	No control group	Strength, mobility, balance, QoL
Callisaya et al. (2021),^25^ Australia, 7/10	RCT, 26 weeks	*n* = 93 (*I* = 44, *C* = 49)	*I* = 73 ± 7 *C* = 73 ± 7	*I* = 61% *C* = 55%	Home	Mild CI, falls, moderate mobility disability, frailty, comorbidities	Usual care + fall education	Strength, mobility, balance, falls
Crotty et al. (2014),^32^ Australia, NA	Feasibility study, 8 weeks	*n* = 104	73 ± 10	42%	Home and aged care facility	Moderate mobility disability, falls, comorbidities	No control group	Implementation measures
Gandolfi et al. (2017),^29^ Italy, 6/10	RCT, 7 weeks	*n* = 76 (*I* = 38 *C* = 38)	*I* = 67 ± 7 *C* = 70 ± 9	*I* = 39% *C* = 26%	Home	PD, falls	In person balance training	Mobility, balance, QoL, implementation measures
Lauzé et al. (2017),^27^ Canada, 5/10	RCT, 12 weeks	*n* = 42 (*I* = 28 *C* = 14)	*I* = 80 ± 8 *C* = 83 ± 7	*I* = 71% *C* = 91%	Aged care facility	Mild to moderate mobility disability, falls, frail, mild CI, comorbidities	Usual care	Strength, mobility, QoL, falls, implementation measures
Li et al. (2021),^26^ USA, 8/10	RCT, 24 weeks	*n* = 30 (*I* = 15 *C* = 15)	*I* = 76 ± 6 *C* = 76 ± 6	*I* = 60% *C* = 80%	Home	Mild CI, falls comorbidities, mild mobility disability	Stretches	Falls, strength, mobility, balance, implementation measures
Mansson et al. (2020),^34^ Sweden, NA	Feasibility study, 16 weeks	*n* = 67 (*I* = 29 *C* = 38)	*I* = 76 ± 5 *C* = 77 ± 3	*I* = 62% *C* = 79%	Home	Falls, balance impairment, mild mobility disability	Otago exercise program booklet	Implementation measures
Taylor et al. (2020),^36^ Australia, NA	Feasibility study, 12 weeks	*n* = 15	83 ± 8	47%	Home	Mild to moderate CI, mobility disability, falls comorbidities	No control group	Strength, mobility, balance, implementation measures
Vestergaard et al. (2013),^28^ Denmark, 6/10	RCT, 20 weeks	*n* = 61 (*I* = 30 *C* = 31)	*I* = 81 ± 3 *C* = 83 ± 4	*I* = 100% *C* = 100%	Home	Moderate mobility disability, comorbidities	Usual care	Strength, mobility, QoL, implementation measures
Wong et al. (2005),^35^ China, NA	Feasibility study, 12 weeks	*n* = 22	75 ± 7	90%	Communtiy center and home	OA, mild mobility disability	No control group	Strength, mobility, balance, QoL, implementation measures

^a^ Physiotherapy Evidence Database (PEDro) Score only for RCTs.^46^

*C*, control; CI, cognitive impairment; *I*, intervention; IQR, interquartile range; mod, moderate; NA, not applicable; OA, osteoarthritis; QoL, quality of life; PD, Parkinson's disease; RCT, randomized controlled trial.

Data are mean ± SD, unless otherwise stated.

### TELEHEALTH AND EXERCISE INTERVENTION COMPONENTS

Telehealth and exercise intervention components are displayed in *Table 2*. Five studies evaluated programs that included synchronous videoconferencing to deliver exercise session,^26,29,31,32,34^ while six studies used asynchronous telehealth to deliver exercise components.^25,27,28,33,35,36^ Seven studies evaluated programs led by a physiotherapist,^25,29,32–36^ six studies incorporated interventions with tailoring of the exercise program,^25,27,29,32,34,35^ and seven studies delivered a combination of strength and balance exercises.^25,27,28,32–35^ Six studies delivered low-intensity exercise,^26,27,29,31,33,36^ with three studies delivering moderate-intensity exercise.^25,34,35^ The median frequency of exercise sessions was three per week (range = 1–7), median session duration was 26 min (range = 7–60), median planned exercise dose over trial was 18 h (range = 3–48), and median intervention length was 17 weeks (range = 4–26). There were three group-based interventions.^26,31,34^

**Table 2.** Telehealth and Exercise Intervention Components (*N* = 11)

REFERENCES	STUDY LEAD	TELEHEALTH	TAILORED	PRIMARY PROFANE EX. CATEGORY	EX. INTENSITY	EX. FREQENCY (SESSIONS/WEEK)	SESSION DURATION (MIN)	EX. DOSE (H)	GROUP OR IND.	TELEHEALTH SUPPORTS
Bernard et al. (2009)^31^	Kinesiologist	Synchronous videoconferencing exercise classes	NR	Strength – seated	Low	1–2 Times/week	60 min	13 h	Group	Local F2F support
Bruns et al. (2019)^33^	PT	Asynchronous exercise videos	No	Strength – seated and standing	Low	7 Times/week	7 min	3 h	Ind.	NR
Callisaya et al. (2021)^25^	PT or EP	Asynchronous exercise videos	Yes	Strength/balance – standing	Moderate	Flexible	10–30 min	48 h	Ind.	One-hour F2F initial ax for setup and 2 FU visits & monthly phone calls
Crotty et al. (2014)^32^	PT	Synchronous videoconferencing	Yes	Strength/balance – standing	NR	2 Times/week	NR	NR	Ind.	Initial F2F training with telehealth
Gandolfi et al. (2017)^29^	PT	Synchronous exercise games	Yes	Balance – standing	Low	3 Times/week	50 min	18 h	Ind.	Remote super-vision & caregiver support
Lauzé et al. (2017)^27^	Kinesiologist	Asynchronous exercise games	Yes	Strength/balance – standing	Low to moderate	2 Times/week	45 min	18 h	Ind.	Initial F2F training with telehealth
Li et al. (2021)^26^	ET	Synchronous videoconferencing exercise class	No	3D ex (Tai Ji Quan) – standing	Low	2 Times/week	60 min	48 h	Group	NR
Mansson et al. (2020)^34^	PT	Asynchronous exercise videos	No	Strength/balance – standing	Low	3 Times/week	30 min	24 h	Ind.	Two hour F2F initial support
Taylor et al. (2019)^36^	PT	Asynchronous exercise videos	Yes	Strength/balance – standing	Moderate	Flexible	10–30 min	17 h	Ind.	F2F initial home visit and 2 FU visits + caregiver support
Vestergaard et al. (2008)^28^	ET	Asynchronous exercise video	No	Strength/balance – standing	NR	3 Times/week	26 min	26 h	Ind.	Initial F2F training, 2 times/week follow-up phone calls
Wong et al. (2005)^35^	PT	Synchronous videoconferencing exercise classes	Yes	Strength/balance – standing	Moderate	4 Times/week (Telehealth 1 time/week)	NR	NR	Group for telehealth	NR

ax, assessment; EP, exercise physiologist; ET, exercise trainer; ex, exercise; F2F, face to face/in person; FU, follow-up; Ind., individual; NR, not reported; PT, physiotherapists.

### EFFECTS OF INTERVENTIONS

**Fig. 2.**
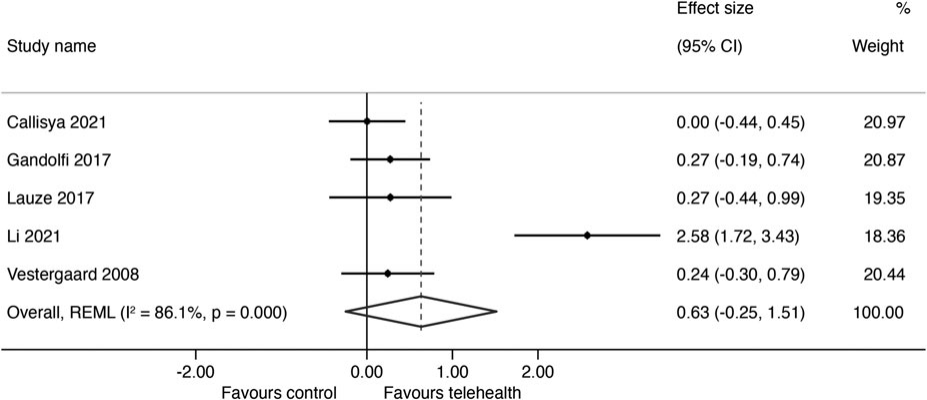
Effect size (95% confidence interval) of telehealth versus control on mobility using random-effects meta-analysis.

**Fig. 3.**
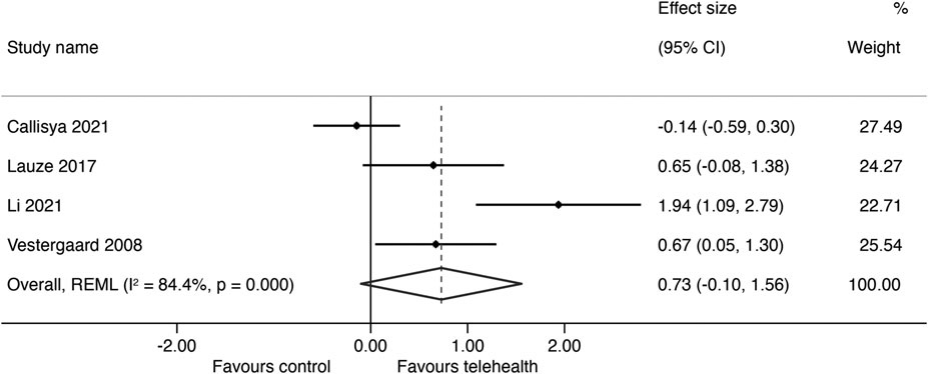
Effect size (95% confidence interval) of telehealth versus control on strength using random-effects meta-analysis.

**Fig. 4.**
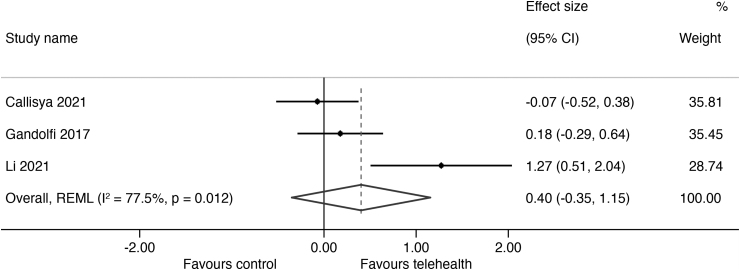
Effect size (95% confidence interval) of telehealth versus control on balance using random-effects meta-analysis.

**Fig. 5.**
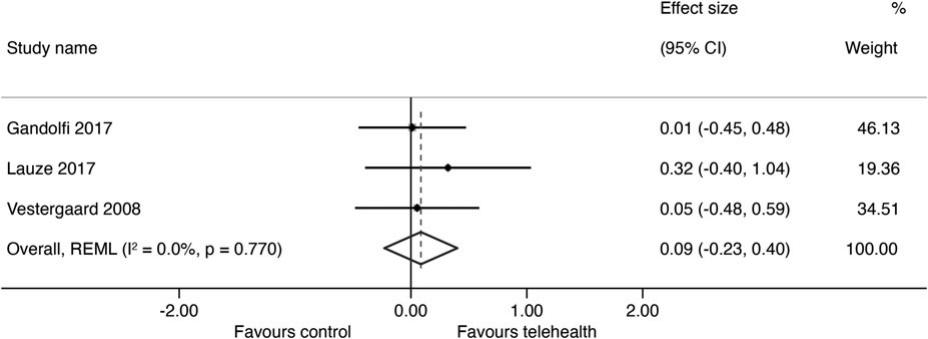
Effect size (95% confidence interval) of telehealth versus control on quality of life using random-effects meta-analysis.

The results of outcomes measured are summarized in *Supplementary Table S2* with the forest plots and mean differences presented in *Figures 2–5*. The overall certainty of evidence for the effect of telehealth intervention on physical outcomes was rated as very low quality (*Supplementary Table S3*) and the pooled results for all outcomes did not reach statistical significance as all the CIs crossed the null line of effect. The pooled estimates (Hedges' g) of suggested effect size are as follows: a moderate improvement in mobility (5 studies; SMD = 0.63; 95% CI = −0.25 to 1.51; 302 participants, *p* = 0.000, *I*^2^ = 86%, very low certainty),^25–29^ a moderate improvement in strength (4 studies; SMD = 0.73; 95% CI = −0.10 to 1.56; 226 participants, *p* = 0.000, *I*^2^ = 84%, very low certainty),^25–28^ and a small improvement in balance (3 studies; SMD = 0.40; 95% CI = −035 to 1.15; 199 participants, *p* = 0.012, *I*^2^ = 78%, very low certainty).^25,26,29^ There was no indication of an effect of telehealth on QoL (3 studies; SMD = −0.09; 95% CI = −0.23 to 0.40; *p* = 0.77, 179 participants, *I*^2^ = 0%, low certainty).^27–29^ We were unable to conduct a meta-analysis of fall outcomes as there were only two studies that could have their reported data translated into a fall rate ratio^27,29^ and only one study was able to translate their fall data into risk ratio.^26^

### IMPLEMENTATION ANALYSIS

The results of the implementation analysis are summarized in *Table 3*.

**Table 3.** Implementation Outcomes

AUTHOR	REACH	FEASIBILITY	ADHERENCE	DOSE ACHIEVED (H)	SAFETY (***N*** = AE)	ACCEPTABILITY (% SATISFIED)
Bernard^31^	NR	67%	57%	10	NR	91%
Bruns^33^	13%	100%	86%	3	NR	80%
Callisaya^25^	69%	83%	85%	41	4	77%
Crotty^32^	51%	92%	NR	NR	NR	NR
Gandolfi^29^	56%	100%	NR	NR	0	91%
Lauzé^27^	NR	79%	89%	16	2	94%
Li^26^	55%	87%	80%	38	0	NR
Mansson^34^	NR	72%	63%	17	0	100%
Taylor^36^	NR	87%	54%	9	1	68%
Vestergaard^28^	9%	87%	89%	23	0	NR
Wong^35^	82%	91%	91%	NR	NR	80%
Total (median) (range)	55% 9–82%	87% 67–100%	85% 54–91%	17 3–41	0 Events 0–4	86% 68–100%

AE, adverse events.

*Reach*: Seven studies reported on the proportion of participants who were successfully screened and consented to participate, and their median reach was 55% (range = 9–82).^25,26,28,29,32,34,36^

*Feasibility:* All studies reported the proportion of participants who were included in the follow-up data collection with a median score of 87% (range = 67–100).

*Adherence:* Nine studies reported a measure of intervention adherence with median exercise attendance to planned exercise sessions as 86% (range = 54–91).^25–28,31,33–36^

*Dose:* Eight studies reported the time spent exercising over the study period with a median exercise dose of 17 h (range = 3–41).^25–28,31,33,35,36^

*Safety:* AE (falls and adverse musculoskeletal pain directly resulted to the telehealth intervention) were reported to some degree in seven studies.^25–29,33,35^ Four studies reported no AE.^26,28,29,33^ Falls (*n* = 4) were reported in three studies from participants exercising, while using asynchronous telehealth.^25,27,35^ One study reported musculoskeletal pain events (*n* = 3) linked to the intervention.^25^

*Acceptability:* Eight trials included a measure of participants' overall satisfaction with the intervention.^25,27,29,31,33–36^ The median satisfaction rate was 86% (range = 68–100).

*Facilitators:* Three factors that supported high program adherence rates were reported by the authors in the “Discussion” section of the article. The importance of initial participant technology training with ongoing support through face-to-face visits or phone calls to troubleshoot any problem and enhance exercise adherence was highlighted by seven authors.^25,27,28,31–33,35^ Participant appreciation of the convenience of telehealth exercise programs being delivered in their own home was reflected on by five authors.^28,29,31,32,36^ Positive effects of program flexibility on exercise adherence using asynchronous telehealth exercise programs as it allowed participants to choose the timing of when they exercised were observed by four authors.^25,27,32,33^

*Barriers:* Authors' reports regarding the impact of the participant's technology hesitation and age on the outcomes of telehealth exercise programs were mixed. One study reported that high technology hesitation reduced recruitment rates.^31^ Two studies observed that higher levels of technology hesitation correlated with reduced telehealth satisfaction rates^26,35^ and another study found that older participants had lower satisfaction rates.^35^ However, one study observed that technology hesitation or age was not related to the feasibility or acceptability of the telehealth intervention.^32^ Bernard et al. commented that delivering synchronous exercise classes to multiple sites is challenging and may inhibit the ability to deliver adequate exercise dose and intensity required to improve other physical outcomes.^31^ Brun et al. stated that asynchronous exercise programs did not enable sufficient exercise tailoring to enhance physical outcomes.^36^

## Discussion

This systematic review with meta-analysis assessed the effectiveness and implementation of exercise interventions delivered through telehealth for older adults, 60 years and older, who are receiving aged care services or have mobility, cognitive, or frailty disability. It is the first review of this approach in this population, to our knowledge. Pooled effects did not reach statistical significance outcomes, but suggested favorable effects of telehealth interventions to improve mobility, strength, and balance in older adults, which are likely to be clinically meaningful. Our analysis of implementation measures suggested that telehealth is feasible, as evidenced by high rates of acceptability and adherence with few safety concerns.

The utilization of telehealth to promote physical activity and improve physical functioning in aged care is emerging as an effective and acceptable mode of health care delivery.^37^ A 2020 systematic review (*n* = 17 controlled studies) into community-dwelling older adults, 60 years of age and older, receiving a variety of health care interventions using synchronous telehealth found similarly high levels of feasibility, safety, and acceptability.^38^ Three of the included studies that focused on falls, exercise, or strength-based measures demonstrated significant improvements.^39–41^ A scoping review in 2022 found that synchronous and asynchronous telehealth physiotherapy is safe, feasible, and acceptable to adults with complex comorbidities and has comparable effects to in-person care activity.^42^ Both reviews suggest that telehealth delivery increases access to exercise programs, especially for those who cannot travel to a treatment facility due to distance or disability.^38,42^ Hawley et al. concluded that telehealth physiotherapy may increase adherence to exercise by providing increased prompts reminding patients when and how to exercise.^42^

The positive effects emerging in our meta-analysis favoring telehealth-led exercise intervention could be explained by their alignment with some behavior change techniques known to facilitate exercise adherence.^43^ Several studies involved elements of monitoring and feedback where they were able to provide real-time feedback on individuals' levels of performance and offered clear instructions on how to perform the exercise.^26,27,29,32^ These elements combined with professional support have been shown to increase exercise adherence.^43^

A set of key components for successful fall prevention exercise programs has been identified in community-dwelling adults.^4^ Interventions that included an exercise dose of more than 3 h per week and included balance and strength exercises reduced falls by 34%.^4^ However, adherence to in-person fall prevention exercise programs has previously been reported to be ∼50%.^44^ In our review, we found that the adherence of exercise interventions delivered by telehealth was high (86%), but the exercise dose achieved was low (1.3 h/week). Future research could investigate how to utilize telehealth's high adherence to support increased exercise dose to improve physical outcomes.

This review highlighted the importance of providing staff and participant training to improve the chances of successful program implementation. The importance of technology training was also reported in a recent qualitative study where telehealth was used to enhance mobility and physical activity for older adults receiving rehabilitation services.^45^ They found that patients engaged optimally with telehealth when they received sufficient training and support to use the technology and understood the potential benefits from using telehealth. Their study also reported the importance of sufficient therapist training to increase telehealth technology competence and overall program implementation.

## Conclusions

This is the first systematic review and meta-analysis that provides a summary of the impact of telehealth on physical outcomes for older adults, 60 years of age and older, with mobility, frailty, and cognitive disabilities. We conducted this systematic review in accordance with PRISMA guidelines and followed a pre-specified protocol registered on PROSPERO. Furthermore, the controlled studies included were of sound methodological quality. However, due to the study heterogeneity and low number of controlled studies in this area, we were unable to uncover any significant finding. There was also insufficient follow-up data, which limited our ability to assess the long-term effects and sustainability of exercise interventions delivered through telehealth in this population.

Future RCTs are required to investigate the use and effects of synchronous different exercise programs delivered through telehealth, trials that investigate use effects of asynchronous telehealth versus asynchronous telehealth, trials that include cost-effective analyses, and trials that explore the implementation and sustainability of these telehealth interventions to ensure that wide scale uptake of telehealth in aged care is as safe, effective, and cost-effective as possible. Telehealth used to deliver evidence-based exercise intervention to our most vulnerable older adults has the potential to be an effective and acceptable addition to in-person exercise interventions.

## Supplementary Material

Supplemental data

Supplemental data

Supplemental data
