# Supplementary material for: Exercise Interventions Delivered Through Telehealth to Improve Physical Functioning for Older Adults with Frailty, Cognitive, or Mobility Disability: A Systematic Review and Meta-Analysis
Source: Telemed J E Health. 2024 Apr 8;30(4):940–50. doi: 10.1089/tmj.2023.0177 (PMC11035924; doi:10.1089/tmj.2023.0177)
Supplement: Supplemental data [file Suppl_TableS1.docx]

**Supplementary Table 1: Search terms for Medline**

| **#** | **Query** | **Results** |
| --- | --- | --- |
| 1 | aged.mp. | 5,804,099 |
| 2 | Aged/ | 3,334,085 |
| 3 | older adults.mp. | 35,090 |
| 4 | older adult*.mp. | 101,558 |
| 5 | elderly.mp. | 283,605 |
| 6 | geriatric.mp. | 84,093 |
| 7 | Geriatrics/ | 30,971 |
| 8 | seniors.mp. | 8,407 |
| 9 | (old* adj2 (men or women or female* or mal* or adult* or adults*)).mp. [mp=title, abstract, original title, name of substance word, subject heading word, floating sub-heading word, keyword heading word, organism supplementary concept word, protocol supplementary concept word, rare disease supplementary concept word, unique identifier, synonyms] | 391,100 |
| 10 | 1 or 2 or 3 or 4 or 5 or 6 or 7 or 8 or 9 | 6,060,642 |
| 11 | fall* prevention.mp. | 4,369 |
| 12 | balance exercise*.mp. | 739 |
| 13 | balance training.mp. | 1,678 |
| 14 | strength exercise*.mp. | 1,003 |
| 15 | strength training.mp. | 6,043 |
| 16 | (strength and balance exercise*).mp. [mp=title, abstract, original title, name of substance word, subject heading word, floating sub-heading word, keyword heading word, organism supplementary concept word, protocol supplementary concept word, rare disease supplementary concept word, unique identifier, synonyms] | 261 |
| 17 | resistance training.mp. | 16,100 |
| 18 | Resistance Training/ | 10,947 |
| 19 | (strength and balance training).mp. [mp=title, abstract, original title, name of substance word, subject heading word, floating sub-heading word, keyword heading word, organism supplementary concept word, protocol supplementary concept word, rare disease supplementary concept word, unique identifier, synonyms] | 445 |
| 20 | 11 or 12 or 13 or 14 or 15 or 16 or 17 or 18 or 19 | 26,012 |
| 21 | telehealth.mp. | 9,761 |
| 22 | telemedicine.mp. | 41,402 |
| 23 | Telemedicine/ | 32,611 |
| 24 | telerehabilitation.mp. | 1,645 |
| 25 | Telerehabilitation/ | 736 |
| 26 | telecare.mp. | 858 |
| 27 | ehealth.mp. | 5,589 |
| 28 | mhealth.mp. | 7,208 |
| 29 | tele-health.mp. | 231 |
| 30 | tele-medicine.mp. | 169 |
| 31 | tele-rehabilitation.mp. | 254 |
| 32 | tele-care.mp. | 36 |
| 33 | e-health.mp. | 3,572 |
| 34 | m-health.mp. | 753 |
| 35 | ((remote* or distant or video) adj3 (consult* or monitor* or treat* or therap*)).mp. [mp=title, abstract, original title, name of substance word, subject heading word, floating sub-heading word, keyword heading word, organism supplementary concept word, protocol supplementary concept word, rare disease supplementary concept word, unique identifier, synonyms] | 20,843 |
| 36 | 21 or 22 or 23 or 24 or 25 or 26 or 27 or 28 or 29 or 30 or 31 or 32 or 33 or 34 or 35 | 70,231 |
| 37 | 10 and 20 and 36 | 111 |
