# Supplementary material for: Exercise Interventions Delivered Through Telehealth to Improve Physical Functioning for Older Adults with Frailty, Cognitive, or Mobility Disability: A Systematic Review and Meta-Analysis
Source: Telemed J E Health. 2024 Apr 8;30(4):940–50. doi: 10.1089/tmj.2023.0177 (PMC11035924; doi:10.1089/tmj.2023.0177)
Supplement: Supplemental data [file Suppl_TableS2.docx]

**Supplementary Table 2: RCT Trial results (n = 5)**

| Author | Outcome | Intervention | | Control | |
| --- | --- | --- | --- | --- | --- |
|  |  | **Baseline** | **Post-trial** | **Baseline** | **Post-trial** |
| Mobility | | | | | |
| Callisaya (25) | *Gait speed (m/sec)* | | | | |
|  |  | 1.3±0.2 | 1.3± 0.2 | 1.22±0.17 | 1..21±5.8 |
| Gandolfi (29) | *10m walk test (m/sec)* | | | | |
|  |  | 1.6±-0.49 | 1.6±0.43 | 1.46±-0.42 | 1.60±1.01 |
| Lauze (27) | *Short Performance Physical Battery test (n/12points)* | | | | |
|  |  | Change | 1.0±1.3 | Change | 0.3±3.6 |
| Li (26) | *Timed Up and Go - single task (m/sec)* | | | | |
|  |  | 13.38±0.55 | 11/86±0.56 | 13.41±0.73 | 13.04±0.89 |
| Vestergaard (28) | *Physical Performance test (n/36)* | | | | |
|  |  | 16.3±5.6 | 18.1±5.8 | 17±4.8 | 17.4±5.5 |
| Strength | | | | | |
| Callisaya (25) | *5 sit to stands (secs)* | | | | |
|  |  | 11.2±4 | 11.9±6.4 | 9.9±3.4 | 9.7±5.8 |
| Lauze (27) | *5 sit to stands (secs)* | | | | |
|  |  | Change | -2.1±2.3 | Change | 0.3±3.6 |
| Li (26) | *30 sec sit to stands (n)* | | | | |
|  |  | 11±1.0 | 13.07±0.8 | 11.4±1.12 | 11.6±1.06 |
| Vestergaard (28) | *5 sit to stands (secs)* | | | | |
|  |  | 19.3±11.6 | 14.1±8.5 | 16.4±5.3 | 16.3±6.2 |
| Balance | | | | | |
| Callisaya (25) | *Step test (n)* | | | | |
|  |  | 17.7±4.0 | 17.59±5.3 | 16.4±3.4 | 15.8±5.8 |
| Gandolfi (29) | *Berg Balance Scale (n/56points)* | | | | |
|  |  | 48.63±6.31 | 52.4±3.29 | 45.61±7.97 | 49.82±1.01 |
| Li (26) | *4 Stage balance test (n/4points)* | | | | |
|  |  | 2.39±0.49 | 3.13±0.52 | 2.47±0.52 | 2.53±0.52 |
| QoL | | | | | |
| Gandolfi (29) | *Parkinson’s Disease Questionnaire (PDQ)-8: lower score higher QoL* | | | | |
|  |  | 30.7±15.5 | 24.2±14.8 | 30.5±16.0 | 24.2±15.9 |
| Lauze (27) | *36 Item Short Form Health Survey (SF-36): higher score higher QoL* | | | |  |
|  |  | Change | -0.7±7.7 | Change | 2.4±6.5 |
| Vestergaard (28) | *EuroQol-visual analogue scales (EQ-VAS): higher score higher QoL* | | | | |
|  |  | 59.8±21 | 59±16.2 | 62.1±17.4 | 60.4±16.7 |
| Falls | | | | | |
| Gandolfi (29) | *Rate* |  |  |  |  |
|  |  | 0.58 | 0.38 | 1.84 | 0.61 |
| Lauze (27) | *Number* |  |  |  |  |
|  |  | NR | 4 | NR | 0 |
| Li (26) | *Number* |  |  |  |  |
|  |  |  | 15 |  | 26 |
| NR=not reported | | | | | |
