# Supplementary material for: Exercise Interventions Delivered Through Telehealth to Improve Physical Functioning for Older Adults with Frailty, Cognitive, or Mobility Disability: A Systematic Review and Meta-Analysis
Source: Telemed J E Health. 2024 Apr 8;30(4):940–50. doi: 10.1089/tmj.2023.0177 (PMC11035924; doi:10.1089/tmj.2023.0177)
Supplement: Supplemental data [file Suppl_TableS3.docx]

**Supplementary Table 3: Summary of the certainty of evidence (GRADE Quality assessment)**

| **Meta-analysis** | **Downgraded** | | | | **Overall** |
| --- | --- | --- | --- | --- | --- |
|  | **Study limitations**^1^ | **Inconsistency^2^** | **Imprecision**^3^ | **Publication bias^4^** |  |
| **Mobility** | - | ↓ (I^2^ = 86%) | ↓ | ↓ | Very Low |
| **Strength** | - | ↓ (I^2^ = 84%) | ↓ | ↓ | Very Low |
| **Balance** | - | ↓ (I^2^ = 78%) | ↓ | ↓ | Very Low |
| **QoL** | - | - (I^2^ = 0%) | ↓ | ↓ | Low |

↓Downgraded

^1^ >25% of participants from studies with low methodologic quality: PEDro score <6

^2^ Heterogeneity > 60%

^3^ Wide confidence intervals around the effect estimate

^4^ Risk of publication bias due to small number of small trials

NB: We did not consider the indirectness criterion in this review as it encompasses a specific population with relevant

outcome measures and direct comparisons
